# Clinical efficacy and adverse effects of LMWH combined with ASA in the treatment of RSA: A meta-analysis and systematic review

**DOI:** 10.1097/MD.0000000000039603

**Published:** 2024-09-13

**Authors:** Fang Liu, Dongmei Shi, Rui Jin, Xinyou Yu

**Affiliations:** a Yinchuan Maternal and Child Health Care Hospital, Yinchuan, Ningxia, China; b General Hospital of Ningxia Medical University, Yinchuan, Ningxia, China.

**Keywords:** ASA, LMWH, meta-analysis, RSA

## Abstract

**Background:**

The incidence of recurrent spontaneous abortion (RSA) in the clinic shows an increasing trend year by year, and the coagulation status of this group of patients is mostly relatively abnormal. Currently, commonly used drugs in clinical practice include Aspirin (ASA) and low molecular weight heparin (LMWH), but their optimal treatment remains controversial. We aimed to evaluate the clinical efficacy and adverse effects of LMWH combined with ASA in the treatment of RSA.

**Methods:**

Randomized controlled trials of LMWH combined with ASA for RSA were searched in the databases of PubMed, EMBASE, Cochrane Library, China National Knowledge Infrastructure, Wanfang, VIP, and Chinese Biomedical Literature Service System from the construction of the database to June 2024. Data were analyzed using Review Manager 5.3 and Stata software. Dichotomous variables were analyzed using relative risk (RR) and 95% confidence interval (CI) as their statistics. The included literature was assessed for bias and risk of bias of eligible studies using Cochrane risk of bias tool. The risk of bias was evaluated based on the evaluation criteria recommended by the Cochrane Guidance Manual for Systematic Evaluation.

**Results:**

A total of 32 papers with a total of 3397 patients with RSA were finally included. LMWH combined with ASA treatment significantly improved the live birth rate (RR = 1.31, 95% CI: [1.19, 1.45], *P* < .00001), the rate of preterm stillbirths (RR = 0.23, 95% CI: [0.13, 0.40], *P* < .00001), rate of term delivery (RR = 1.55, 95% CI: [1.43, 1.67], *P* < .00001), rate of miscarriage (RR = 0.42, 95% CI: [0.36, 0.48], *P* < .00001), incidence of petechiae (RR = 0.44, 95% CI: [0.26, 0.72], *P* = .001), and incidence of thrombocytopenia (RR = 0.61, 95% CI: [0.39, 0.96], *P* = .03). In contrast, the incidence of preterm live births (RR = 1.07, 95% CI: [0.90, 1.28], *P* = .44), adverse reactions (RR = 0.77, 95% CI: [0.59, 1.00], *P* = .05), gingival bleeding (RR = 1.12, 95% CI: [0.65, 1.93], *P* = .69), and gastrointestinal reactions (RR = 0.87, 95% CI: [0.64, 1.17], *P* = .35) were not significant.

**Conclusion:**

LMWH combined with ASA treatment might improve pregnancy outcomes and reduces the incidence of adverse events in patients with RSA.

## 1. Introduction

Recurrent spontaneous abortion (RSA) is one of the clinical infertility that seriously affects women’s reproductive health, and its definition varies from country to country,^[1,2]^ and in China, it is defined as 2 or more consecutive occurrences of fetal loss with the same partner, before the 28th week of gestation.^[3]^ Epidemiologic studies have shown that the incidence of RSA in pregnant women is approximately 1% to 5%,^[4]^ and the risk of RSA increases with the number of miscarriages. It is generally accepted that patients with a history of 3 or more abortions have a risk of reabortion of up to 40% or more.^[3]^ The etiology of RSA is complex and varied, and is associated with chromosomal or genetic abnormalities, anatomical abnormalities, autoimmune disorders, pre-thrombotic states (prothrombotic), endocrine abnormalities, infectious factors, male factors, environmental factors, and psychological factors, with the majority of RSA occurring for unknown reasons.^[3,5]^

RSA not only triggers symptoms such as embryonic arrest and biochemical pregnancy, but also has a serious impact on an individual’s physical and mental health if not intervened in a timely manner. Therefore, the clinic needs to actively explore effective solutions for the treatment of patients. Although Aspirin (ASA) is effective in the treatment of RSA, the rate of reabortion is still high. low molecular weight heparin (LMWH) is a kind of anticoagulant drug, and a number of studies have confirmed that it has a good application effect in the treatment of RSA.^[3]^

Some studies have suggested that ASA combined with LMWH is superior to ASA alone in the treatment of RSA,^[6]^ while a study by Kaandorp SP et al^[7]^ found that there was no significant difference between the use of ASA combined with LMWH and the application of ASA alone in the treatment of RSA when comparing its live birth rate, which has a high incidence of RSA in the clinic, but the optimal treatment of it is still controversial. Therefore, this study used meta-analysis to comprehensively and systematically evaluate the clinical efficacy and adverse effects of ASA combined with LMWH in the treatment of RSA.

## 2. Methods

This meta-analysis was produced following the Preferred Reporting Items for Systematic Reviews and Meta-Analyses (PRISMA) statement.

### 2.1. Literature search strategy

The study followed the Cochrane principles and searched PubMed, EMBASE, Cochrane Library, China National Knowledge Infrastructure, Wanfang, VIP, and Chinese Biomedical Literature Service System, with a time frame from the establishment of the database to June 2024, respectively. The search terms were “Low Molecular Weight Heparin,” “Aspirin,” “Habitual Abortion.” The search language types mainly included Chinese and English, and other languages were not included in this study.

### 2.2. Inclusion criteria

Type of study: randomized controlled trial (RCT).

Study population: RSA patients.

Intervention: control group treated with ASA; experimental group treated with ASA combined with LMWH.

Endpoints: Live birth, preterm live birth, preterm stillbirth, full-term delivery, miscarriage, petechiae and gingival bleeding, thrombocytopenia, gastrointestinal reactions, and other adverse effects.

### 2.3. Exclusion criteria

Patients with pre-thrombotic state, animal experimental studies, prospective studies, cohort experimental studies, reviews, and other types of literature were excluded from this study.

### 2.4. Literature screening and data extraction

The 2 researchers screened the literature independently with strict reference to the inclusion and exclusion criteria, cross-checked, and resolved any disagreement through discussion within the group or according to the discussion of the third researcher, and finally determined the inclusion of the literature. A “data extraction form” was developed, which included: authors of the included studies, year of publication, number of experimental and control groups, treatment measures in experimental and control groups, and main outcome data.

### 2.5. Risk of bias assessment

Two researchers evaluated the quality of the included studies based on the evaluation criteria recommended by the Cochrane Guidance Manual for Systematic Evaluation.^[8]^ The tool included the following components: (1) generation of the randomized allocation scheme; (2) concealment of the allocation sequence; (3) implementation of blinding for all investigators and subjects; (4) implementation of blinding for outcome assessment; (5) completeness of data results; (6) selective reporting of results; and (7) other sources of bias. Each risk of bias level was categorized as low, high, or unclear, and results were represented using different color blocks with corresponding risk of bias plots. In case of disagreement during the assessment process, a third researcher’s results were used for the final assessment. GRADE assessment was used to access the quality of evidence.

### 2.6. Statistical analysis

In this study, dichotomous variables were analyzed for outcomes using relative risk (RR) and 95% confidence interval (CI) as their statistics. Heterogeneity analysis of the studies was assessed using the I^2^ statistic value as well as the χ^2^ test, and when the statistical heterogeneity among the studies was small (*P* ≥ .10, I^2^ ≤ 50%), a fixed-effects model was used; conversely, when the statistical heterogeneity among the studies was large (*P* < .10, I^2^ > 50%), a random-effects model was used, and sensitivity analysis was performed to look for the source of the heterogeneity, or, if the heterogeneity was too large, a further subgroup analysis was performed. Forest plots and funnel plots were created using Review Manager 5.3 software. Statistical analysis was performed using STATA 15.1 software. Differences were considered statistically significant when *P* < .05.

## 3. Results

### 3.1. Search results and evaluation of literature quality

**Figure 1.**
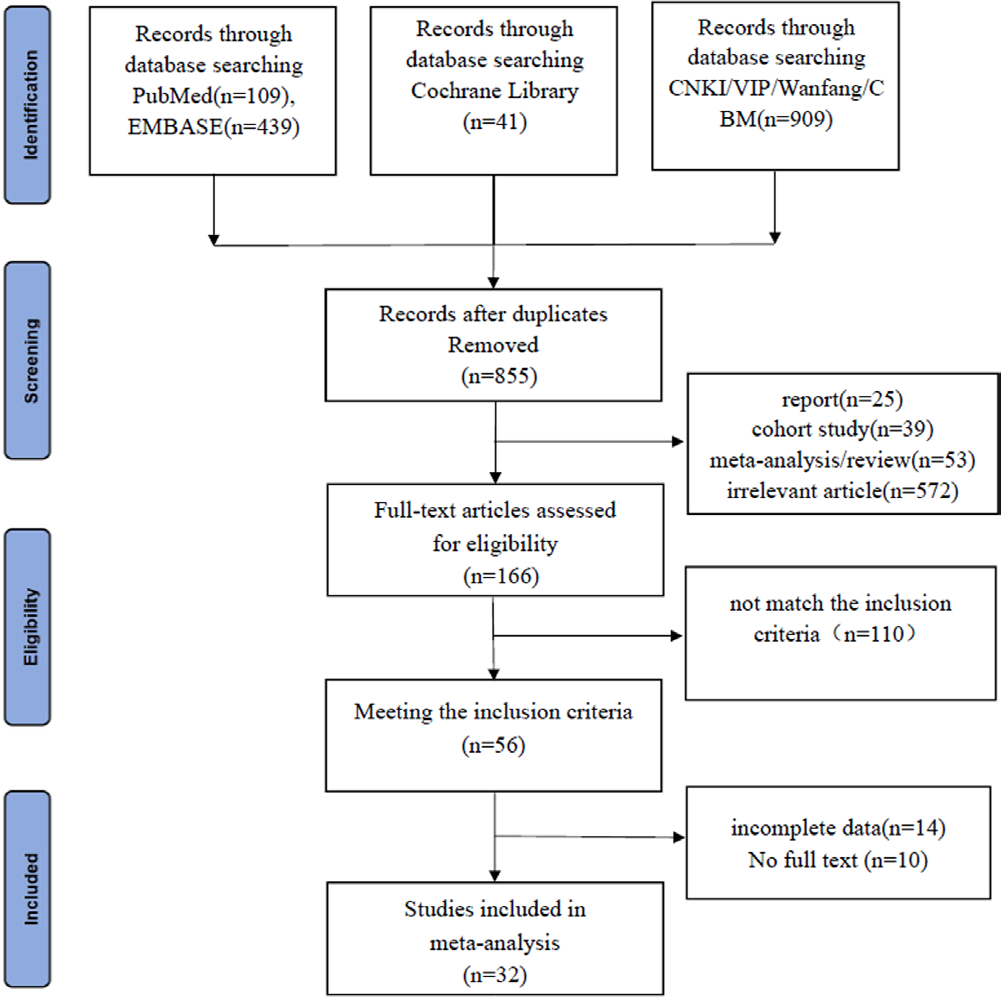
Literature screening flowchart.

Literature search was conducted according to the developed search strategy, a total of 1498 documents were obtained from the initial search of this study, 855 documents were obtained after de-emphasis, 689 documents were excluded by reading the title and the abstract of the literature, and finally 32 RCTs were included. The literature screening process is shown in Figure 1.

### 3.2. Basic characteristics of the included studies

A total of 32 clinical studies were included in this study.^[6,7,9–38]^ The basic characteristics of the included studies and the basic information of the patients are detailed in Table 1.

**Table 1:**
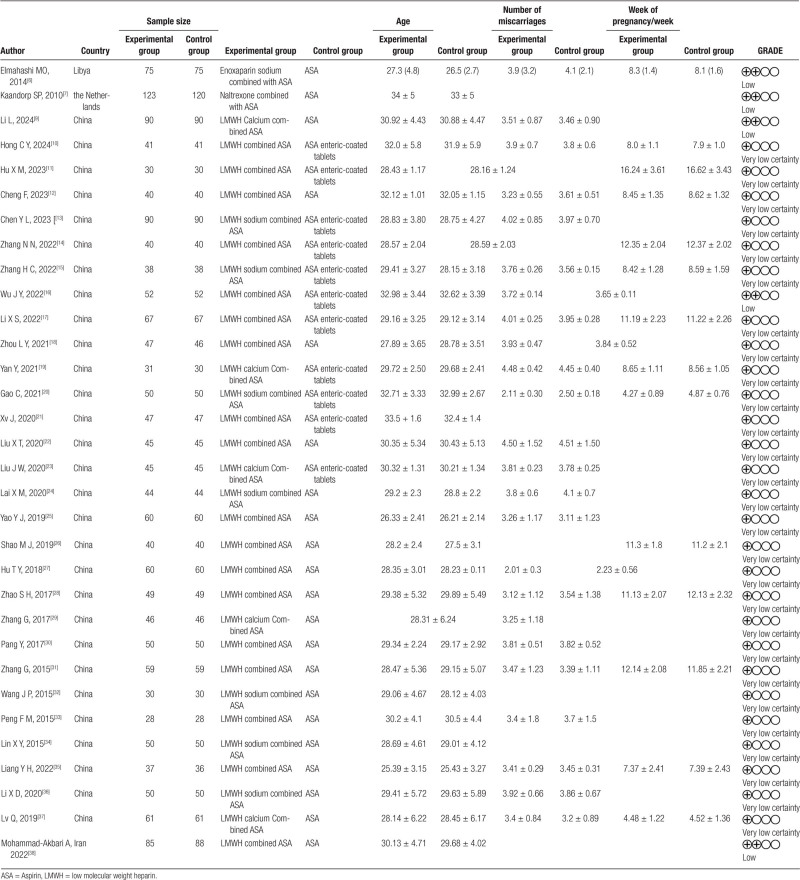
Basic characteristics of included studies.

### 3.3. Risk of bias assessment

**Figure 2.**
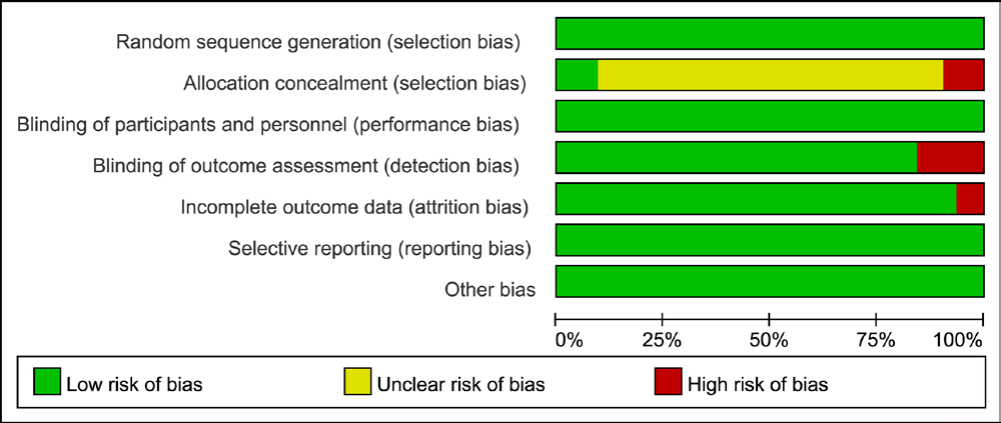
Risk of bias graph.

**Figure 3.**
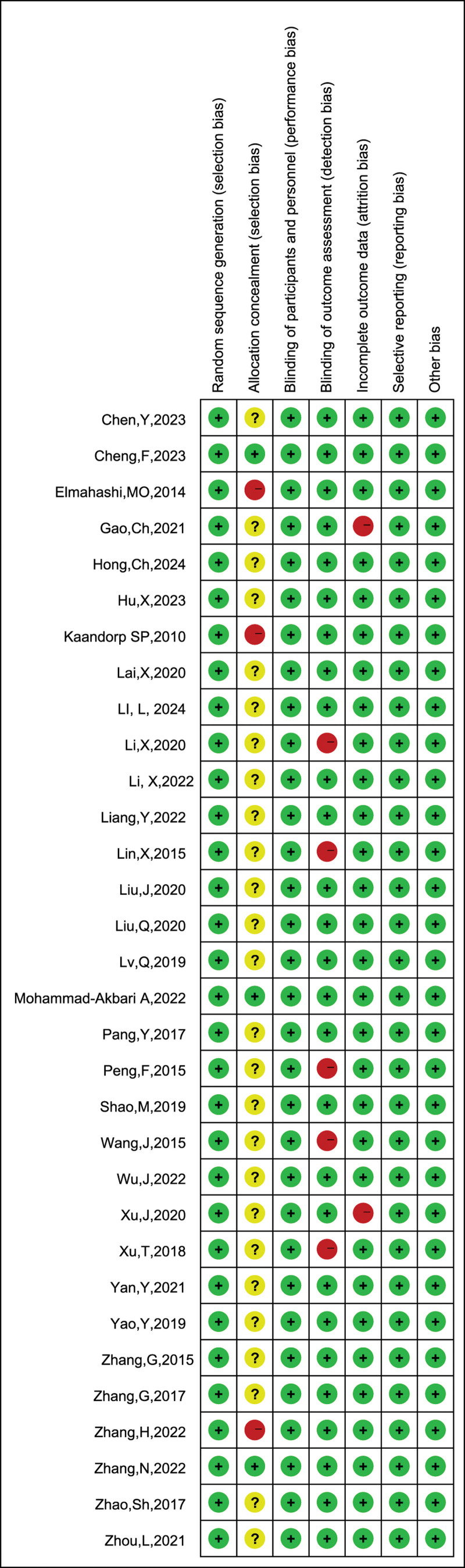
Overview of risk of bias.

The risk of bias maps of the included studies are shown in Figures 2 and 3. The vast majority of the studies in the literature included in this study had a low risk of bias, with 10 containing high risk of bias sub-risk outcomes, and 26 studies were unspecified as to whether or not they were in a covert grouping.

### 3.4. Results of meta-analysis

#### 3.4.1. Pregnancy outcome indicators

##### 3.4.1.1. Live births

**Figure 4.**
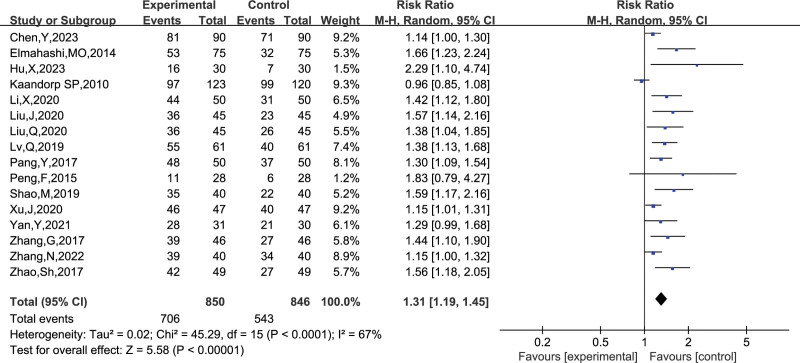
Forest plot of the effect of LMWH combined with ASA treatment on live birth rate in patients with RSA.

A total of 16 studies reported the number of live births, and combining the data revealed heterogeneity among studies (I^2^ = 67%, *P* < .0001), so a random-effects model was chosen to analyze this outcome indicator. The results in Figure 4 show that LMWH combined with ASA treatment improved the live birth rate in patients with RSA compared to controls (RR = 1.31, 95% CI: 1.19, 1.45).

##### 3.4.1.2. Preterm live births

**Figure 5.**
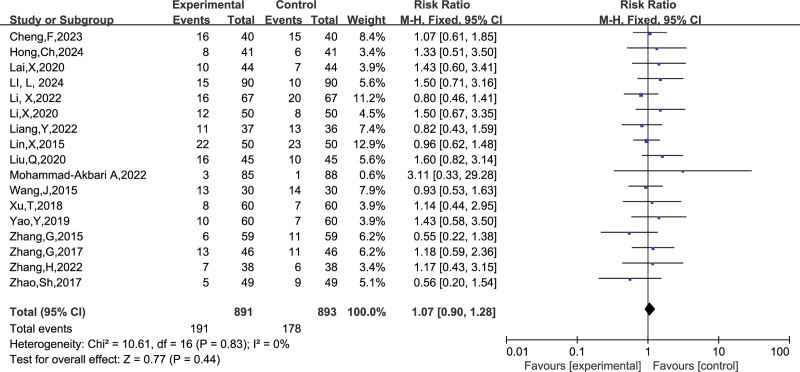
Forest plot of the effect of LMWH combined with ASA treatment on preterm live births in patients with RSA.

**Figure 6.**
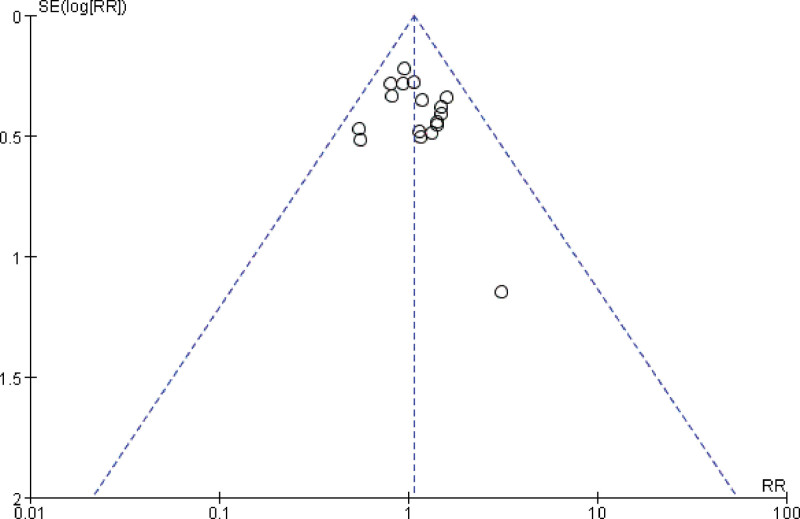
Funnel plot of the effect of LMWH combined with ASA treatment on preterm live births in patients with RSA.

A total of 17 studies reported the number of preterm live births, and combining the data revealed no heterogeneity between studies (I^2^ = 0%, *P* = .83), so a fixed-effects model was chosen to analyze this outcome indicator. The results in Figure 5 show that treatment with LMWH combined with ASA versus ASA alone had no significant effect on improving the rate of preterm live births in patients with RSA compared with controls (RR = 1.07, 95% CI: 0.90 1.28). The funnel plot results in Figure 6 show that there is no publication bias in this result.

##### 3.4.1.3. Preterm stillbirths

**Figure 7.**
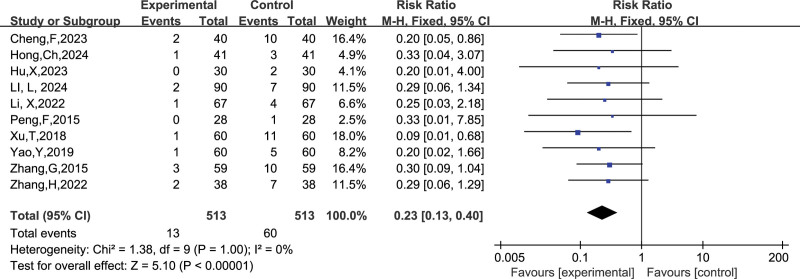
Forest plot of the effect of LMWH combined with ASA treatment on preterm stillbirths in patients with RSA.

**Figure 8.**
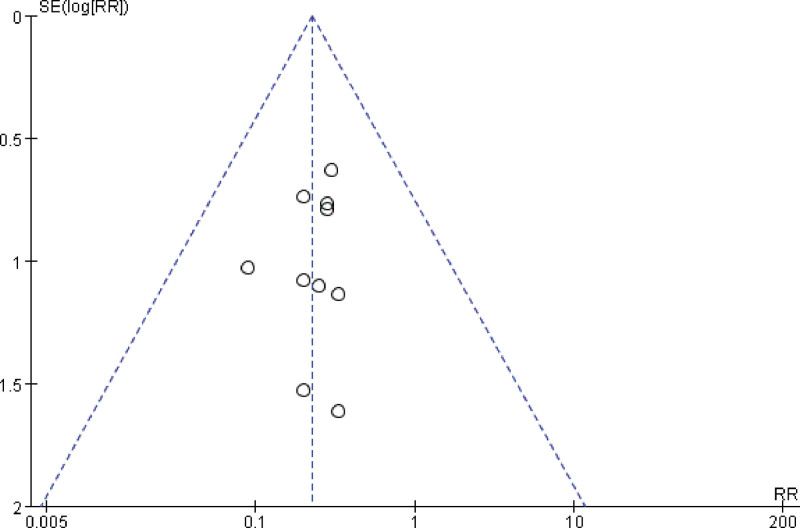
Funnel plot of the effect of LMWH combined with ASA treatment on preterm stillbirth in patients with RSA.

A total of 10 studies reported the number of preterm stillbirths, and after combining the data it was found that there was no heterogeneity between studies (I^2^ = 0%, *P* = 1.00), so a fixed-effects model was chosen to analyze this outcome indicator. The results in Figure 7 show that LMWH combined with ASA improved the rate of preterm stillbirths in patients with RSA compared to controls (RR = 0.23, 95% CI: 0.13 0.40). The funnel plot results in Figure 8 show that there is no publication bias in this result.

##### 3.4.1.4. Full-term births

**Figure 9.**
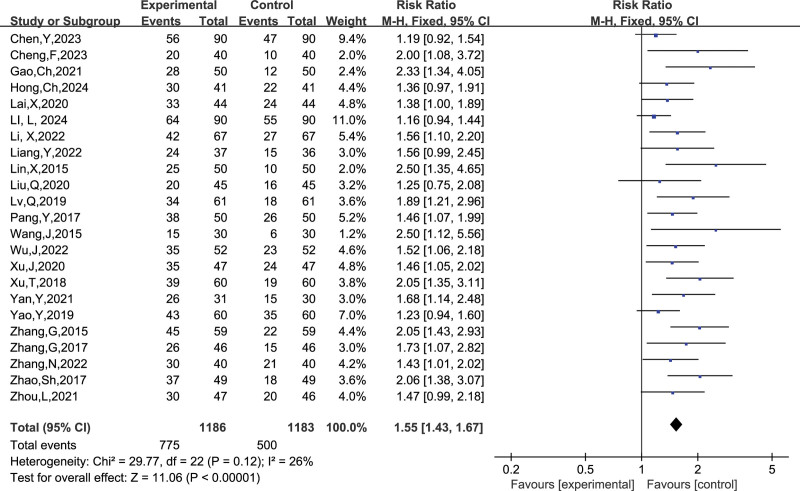
Forest plot of the effect of LMWH combined with ASA treatment on full-term labor in patients with RSA.

**Figure 10.**
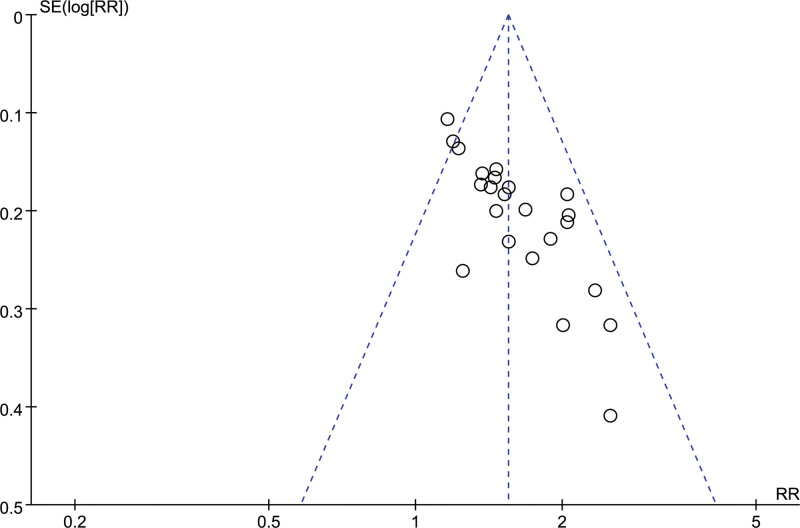
Funnel plot of the effect of LMWH combined with ASA therapy on full-term labor in patients with RSA.

A total of 23 studies reported the number of full-term deliveries, and combining the data revealed small heterogeneity between studies (I^2^ = 26%, *P* = 1.00), so a fixed-effects model was chosen to analyze this outcome indicator. The results in Figure 9 show that LMWH combined with ASA improved the rate of full-term deliveries in patients with RSA compared with controls (RR = 1.55, 95% CI: 1.43, 1.67). The results of the funnel plot in Figure 10 show an asymmetrical left-right distribution of the study sites, suggesting some publication bias, which may be related to the inconsistent timing of drug administration, for example.

##### 3.4.1.5. Miscarriages

**Figure 11.**
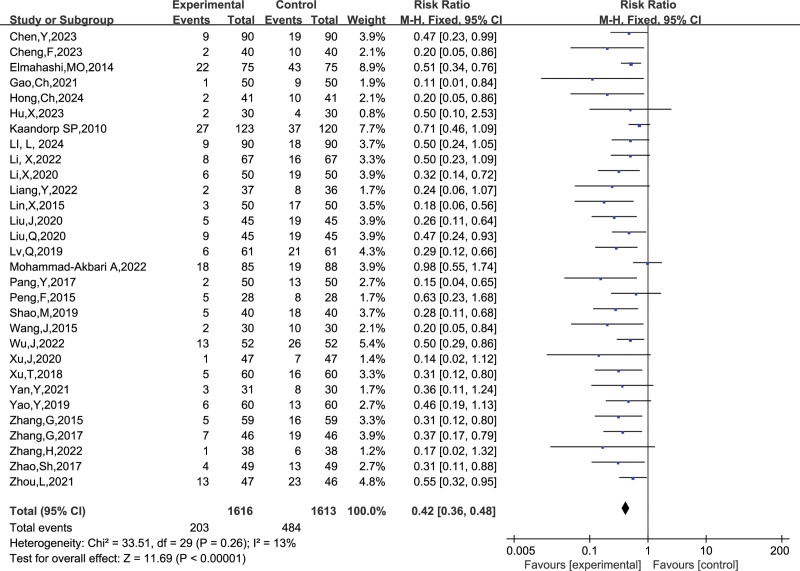
Forest plot of the effect of LMWH combined with ASA treatment on abortion rates in patients with RSA.

**Figure 12.**
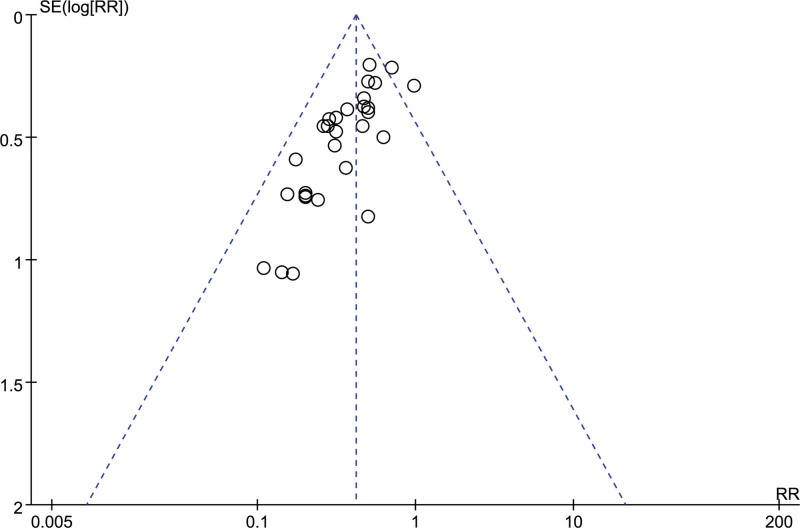
Funnel plot of the effect of LMWH combined with ASA treatment on abortion rates in patients with RSA.

A total of 30 studies reported the number of miscarriages, and combining the data revealed a small heterogeneity between studies (I^2^ = 13%, *P* = .26), so a fixed-effects model was chosen to analyze this outcome indicator. The results in Figure 11 show that LMWH combined with ASA improved the miscarriage rate in patients with RSA compared to controls (RR = 0.42, 95% CI: 0.36 0.48). The results of the funnel plot in Figure 12 show an asymmetrical left-right distribution of the study sites, suggesting some publication bias, which may be related to inconsistencies in the dose administered, the time of administration, and so on.

#### 3.4.2. Safety indicators

##### 3.4.2.1. Adverse reactions

**Figure 13.**
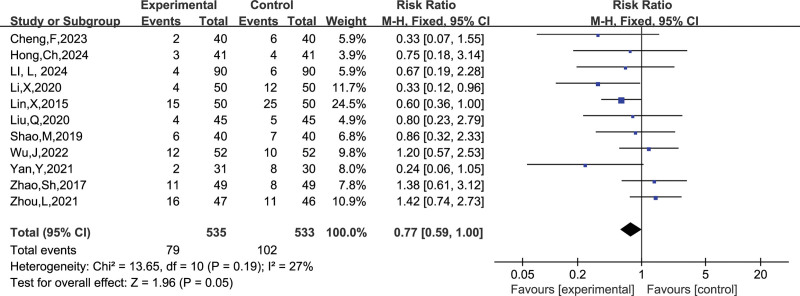
Forest plot of LMWH combined with ASA therapy on the occurrence of adverse effects in patients with RSA.

**Figure 14.**
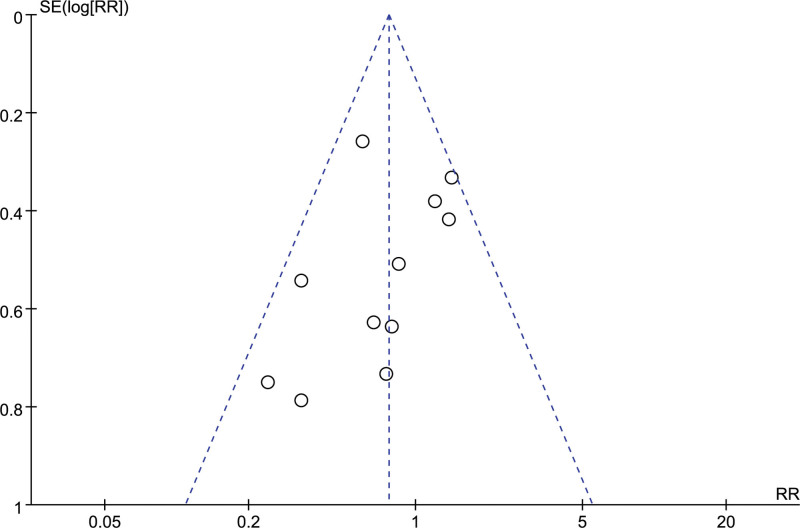
Funnel plot of the occurrence of adverse effects of LMWH combined with ASA therapy in patients with RSA.

A total of 11 studies reported the number of people who experienced adverse reactions, and combining the data revealed a small heterogeneity between studies (I^2^ = 27 %, *P* = .19), so a fixed-effects model was chosen to analyze this outcome indicator. The results in Figure 13 show no significant effect of LMWH combined with ASA on the incidence of adverse reactions in RSA patients compared with controls (RR = 0.77, 95% CI: 0.59 1.00). The funnel plot results in Figure 14 show that there is no publication bias in this result.

##### 3.4.2.2. Ecchymosis

**Figure 15.**
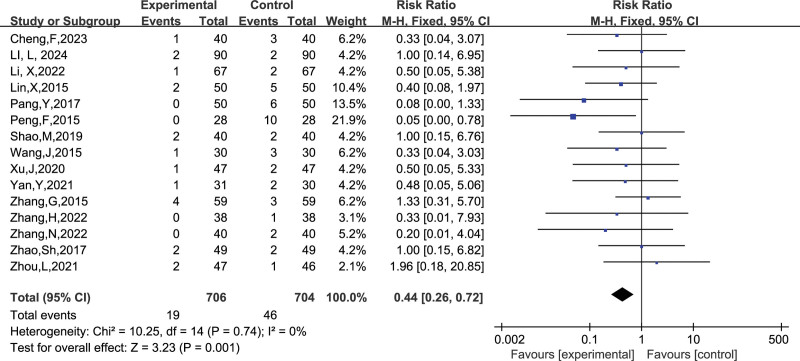
Forest plot of the effect of LMWH combined with ASA therapy on the incidence of petechiae in patients with RSA.

**Figure 16.**
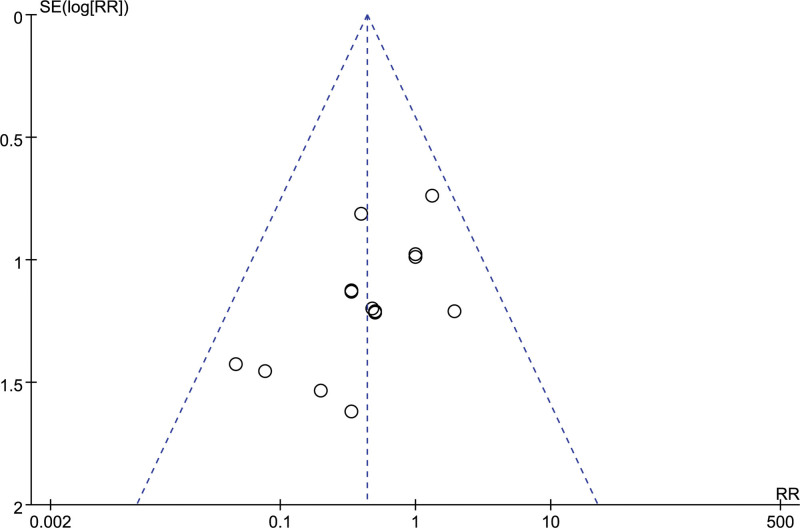
Funnel plot of the effect of LMWH combined with ASA therapy on the incidence of petechiae in patients with RSA.

A total of 15 studies reported the number of people who developed petechiae, and combining the data revealed no heterogeneity between studies (I^2^ = 0%, *P* = .74), so a fixed-effects model was chosen to analyze this outcome indicator. The results in Figure 15 show that LMWH combined with ASA improved the incidence of petechiae in patients with RSA compared with controls (RR = 0.44, 95% CI: 0.26 0.72). The funnel plot results in Figure 16 show that there is no publication bias in this result.

##### 3.4.2.3. Gingival bleeding

**Figure 17.**
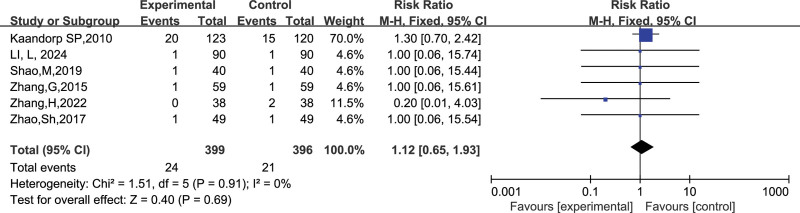
Forest plot of the effect of LMWH combined with ASA treatment on the incidence of gingival bleeding in patients with RSA.

A total of 6 studies reported the number of gingival bleeding, and after combining the data, it was found that there was no heterogeneity among the studies (I^2^ = 0 %, *P* = .91), so a fixed-effect model was chosen to analyze this outcome indicator. The results in Figure 17 show no significant effect of LMWH combined with ASA on the incidence of gingival bleeding in RSA patients compared to controls (RR = 1.12, 95% CI: 0.65, 1.93).

##### 3.4.2.4. Thrombocytopenia

**Figure 18.**
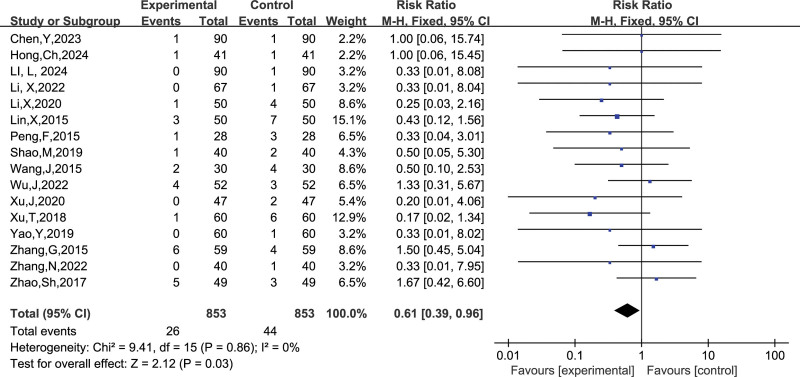
Forest plot of the effect of LMWH combined with ASA therapy on the incidence of thrombocytopenia in patients with RSA.

**Figure 19.**
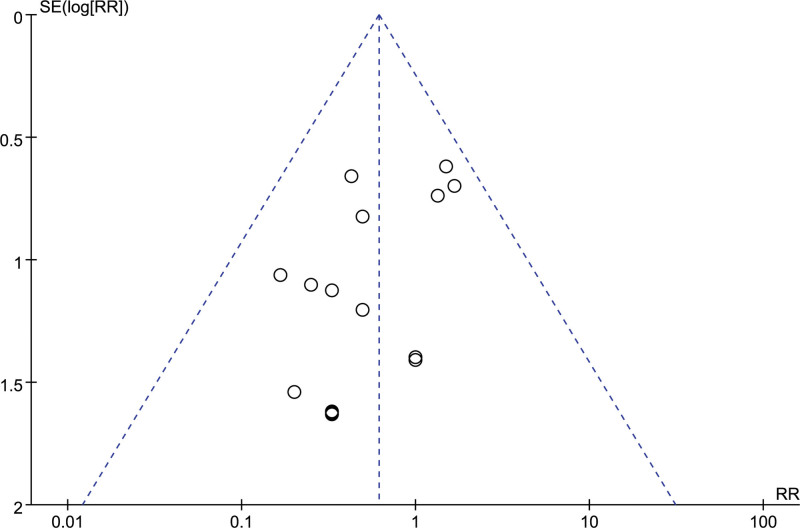
Funnel plot of the effect of LMWH combined with ASA therapy on the incidence of thrombocytopenia in patients with RSA.

A total of 16 studies reported the number of thrombocytopenia, and after combining the data it was found that there was no heterogeneity between studies (I^2^ = 0 %, *P* = .86), so a fixed-effects model was chosen to analyze this outcome indicator. The results in Figure 18 show that LMWH combined with ASA reduced the incidence of thrombocytopenia in patients with RSA compared to controls (RR = 0.61, 95% CI: 0.39, 0.96). The funnel plot results in Figure 19 show that there is no publication bias in this result.

##### 3.4.2.5. Gastrointestinal reactions

**Figure 20.**
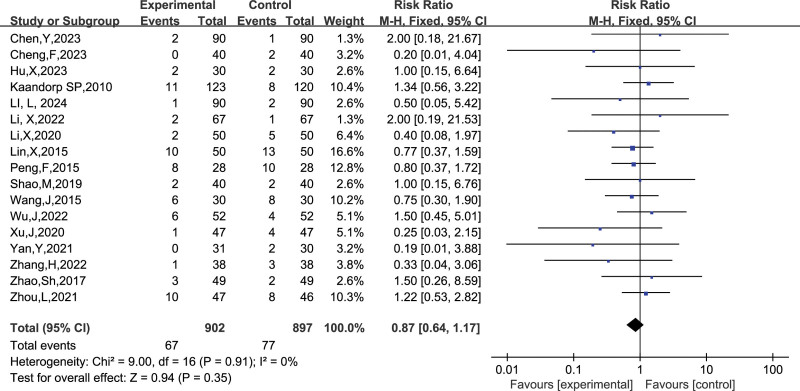
Forest plot of the effect of LMWH combined with ASA therapy on the incidence of gastrointestinal reactions in patients with RSA.

**Figure 21.**
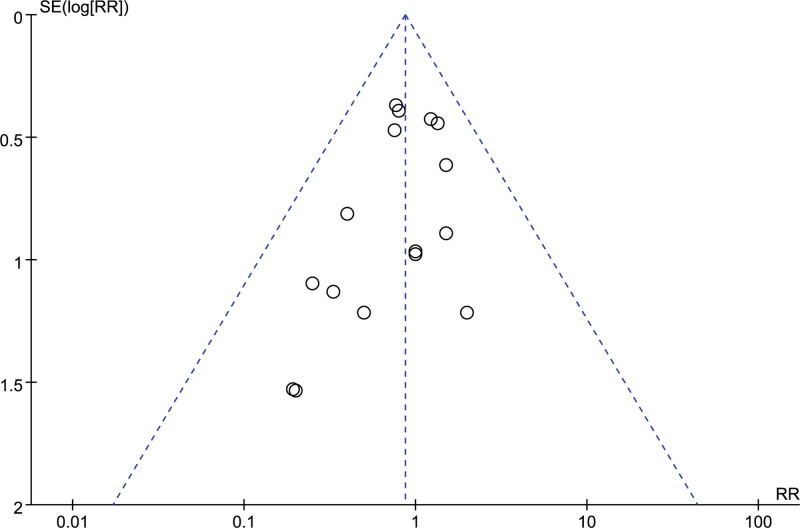
Funnel plot of the effect of LMWH combined with ASA therapy on the incidence of gastrointestinal reactions in patients with RSA.

A total of 17 studies reported the number of gastrointestinal reactions, and after combining the data it was found that there was no heterogeneity between studies (I^2^ = 0 %, *P* = .91), so a fixed-effects model was chosen to analyze this outcome indicator. The results in Figure 20 show no significant effect of LMWH combined with ASA on the incidence of gastrointestinal reactions in patients with RSA compared to controls (RR = 0.87, 95% CI: 0.64, 1.17). The funnel plot results in Figure 21 show that there is no publication bias in this result.

##### 3.4.2.6. Sensitivity analysis

In this study, a one-way sensitivity analysis was conducted on the live birth rate, an outcome indicator with large heterogeneity, and the results showed that the combined effect values were relatively close before and after the exclusion of any one study, suggesting that the results of this study were stable.

## 4. Discussion

With the increase of people’s life and work pressure, the incidence of RSA in the clinic shows a rising trend year by year, and its pathogenesis is very complex, which may be related to a variety of factors, and it is currently believed that the miscarriage-related genes are mainly related to the maternal coagulation function, immune metabolism, and embryo implantation, growth and development, which need to be further researched.^[3]^

Drugs are the first choice for the treatment of RSA, of which ASA and LMWH are commonly used. ASA is a derivative of salicylic acid, an antipyretic and analgesic drug with both antithrombotic and anti-inflammatory effects, which can inhibit the synthesis of thromboxane A2 and thus prevent platelet aggregation, and play an antithrombotic role. Treatment with ASA can reduce the risk of miscarriage and improve the live birth rate of RSA, but the reproductive prognosis of ASA alone is poor due to the many causes of RSA in women during pregnancy. LMWH belongs to a kind of anticoagulant, which is commonly used in the prevention and treatment of venous thrombosis, with multiple effects of anticoagulation, pro-fibrinolysis, protection of phospholipids, immunomodulation, and promotion of trophoblast proliferation and differentiation, and it does not affect the fetus, but the effect of a single application of LMWH is also general.^[19]^

Currently there is a lack of targeted diagnostic and therapeutic programs for RSA, and clinical outcomes are poor. Previous studies have shown inconsistent results, with some studies suggesting that ASA combined with LMWH is superior to ASA alone in the treatment of RSA,^[6]^ and Li J et al^[39]^ showed that ASA combined with LMWH in the treatment of RSA significantly increased the rate of live births compared to ASA alone. The study by Kaandorp SP et al^[7]^ found no significant difference in the live birth rate when comparing the use of ASA combined with LMWH with ASA alone in the treatment of RSA and the results of the study published by Tong L et al^[40]^ also showed that ASA combined with heparin did not improve the live birth rate of the patients when compared with the placebo group but the safety profile was relatively better. However, fewer RCTs were included in the above studies, and the conclusions were inconsistent among the studies, and their optimal treatments are still controversial; therefore, this study used meta-analysis to comprehensively and systematically evaluate the efficacy and safety of ASA combined with LMWH in the treatment of RSA. The results found that LMWH combined with ASA treatment significantly improved the rates of live births, preterm stillbirths, full-term deliveries, and miscarriages in patients with RSA, and no more adverse effects occurred.

Although this study provides a reference for the clinical use of ASA in combination with LMWH in the treatment of RSA, there are some limitations. Firstly, efforts have been made to include more and fuller RCT studies in this study, but the quality of the literature is low and the sample size is small, which may introduce a bias in the study effect. Secondly, the dosage and duration of co-administration varied between studies, but the differences were too large for between-group analysis, which may have an impact on the generalizability of the results.

In summary, the results of this study found that LMWH combined with ASA therapy improved pregnancy outcomes and reduced the incidence of adverse events in patients with RSA. However, due to the lack of a multicenter, high-quality RCT, no final conclusions can be made yet, and further studies are still needed to provide stronger evidence.

## Author contributions

**Conceptualization:** Fang Liu.

**Data curation:** Fang Liu, Dongmei Shi, Rui Jin, Xinyou Yu.

**Formal analysis:** Xinyou Yu.

**Project administration:** Dongmei Shi, Rui Jin.

**Writing – original draft:** Fang Liu, Dongmei Shi, Rui Jin, Xinyou Yu.

**Writing – review & editing:** Fang Liu, Dongmei Shi, Rui Jin, Xinyou Yu.
